# Glial modulation of synapse development and plasticity: oligodendrocyte precursor cells as a new player in the synaptic quintet

**DOI:** 10.3389/fcell.2024.1418100

**Published:** 2024-08-27

**Authors:** Yetunde O. Akinlaja, Akiko Nishiyama

**Affiliations:** ^1^ Department of Physiology and Neurobiology, University of Connecticut, Storrs, CT, United States; ^2^ Institute of Brain and Cognitive Sciences, University of Connecticut, Storrs, CT, United States; ^3^ Institute of Systems Genomics, University of Connecticut, Storrs, CT, United States

**Keywords:** synapse, plasticity, glia, astrocyte, microglia, oligodendrocyte precursor cell, NG2, neuronal activity

## Abstract

Synaptic communication is an important process in the central nervous system that allows for the rapid and spatially specified transfer of signals. Neurons receive various synaptic inputs and generate action potentials required for information transfer, and these inputs can be excitatory or inhibitory, which collectively determines the output. Non-neuronal cells (glial cells) have been identified as crucial participants in influencing neuronal activity and synaptic transmission, with astrocytes forming tripartite synapses and microglia pruning synapses. While it has been known that oligodendrocyte precursor cells (OPCs) receive neuronal inputs, whether they also influence neuronal activity and synaptic transmission has remained unknown for two decades. Recent findings indicate that OPCs, too, modulate neuronal synapses. In this review, we discuss the roles of different glial cell types at synapses, including the recently discovered involvement of OPCs in synaptic transmission and synapse refinement, and discuss overlapping roles played by multiple glial cell types.

## 1 Introduction

**FIGURE 1:**
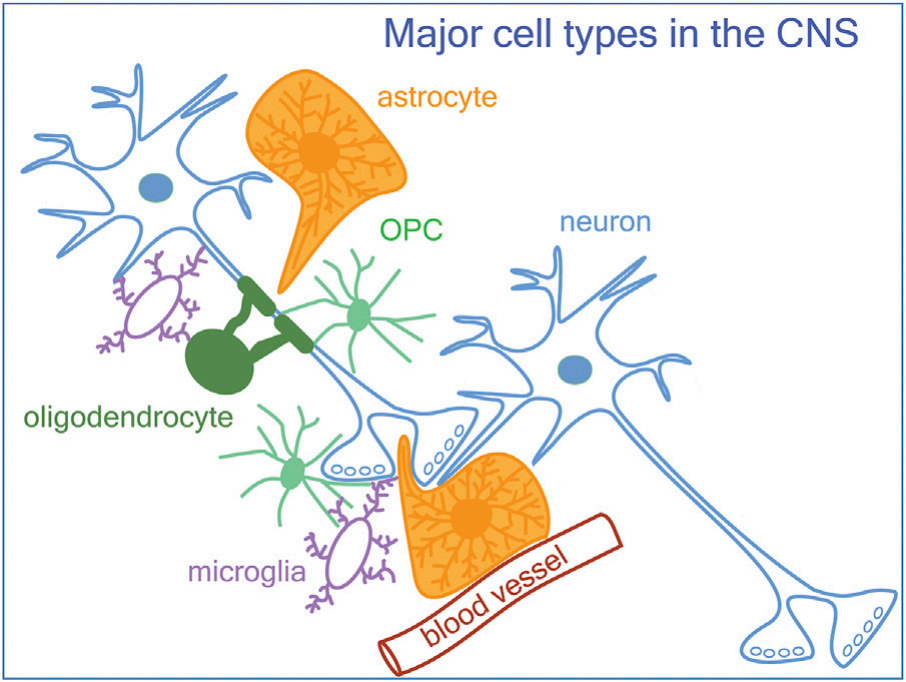
A schematic of neurons, glia, and the vasculature in the central nervous system. In addition to astrocytes and microglia known to be involved in synapse formation and remodeling, OPCs (oligodendrocyte precursor cells) also modulate synapses.

The function of the nervous system is to achieve a point-to-point transmission of information, which differs from other modes of communication such as endocrine and paracrine communication in that it allows for exquisite spatial and temporal specificity and precision. The basic unit of this mode of communication is the chemical synapse, which consists of an elaboration of specific morphological and biochemical machinery at the presynaptic terminal and the adjacent postsynaptic membrane that enables fast and spatially restricted transmission of signals. Each neuron receives a multitude of synaptic inputs at different subcellular compartments, and their net effect on the membrane potential is integrated to bring about a specific level of depolarization at the axon hillock, which determines whether the neuron will generate an action potential. The proper function of the nervous system depends on the coordinated firing of excitatory principal neurons within a neural circuit. The classical view posited that information transfer occurs across individual neurons, and that the regulation of neurons in the network is mediated exclusively by neurons. However, emerging evidence indicates that non-neuronal elements in the central nervous system (CNS) play major roles in modulating neuronal excitability, synaptic transmission, and network function. Here we review some of the roles of different glial cell types at the synapse, including those of oligodendrocyte precursor cells (OPCs), the new kid on the block in synapse regulation (Figure 1). Multiple glial cell types affect the same type of synapses. While the nature of the cross-talk between between different glial cell types at the synapse still remains largely unknown, we provide examples of overlapping roles and transcriptomic profiles for multiple glial cell types, which suggest potential cross-talk between these glia at the synapse.

## 2 Cellular constituents in the CNS form the environment in which neurons function

In the CNS, neurons, astrocytes, oligodendrocytes (OLs), and OPCs develop from a common neurectoderm, while microglia infiltrate and take permanent residence in the CNS in mid-gestation. These resident cells modulate synapse development, plasticity, and refinement.

### 2.1 Astrocytes

Astrocytes are generated from neural progenitor cells in the germinal zones following neurogenesis and continue to proliferate until the first postnatal week. During the ensuing postnatal period, they dramatically increase their morphological complexity and eventually occupy non-overlapping domains that cover the entire CNS parenchyma. Protoplasmic astrocytes in the gray matter have functional microdomains with independently regulated Ca^2+^ transients (Grosche et al., 1999; Bushong et al., 2002). Each of the microdomains ensheaths 300–600 dendrites (Halassa et al., 2007). Three-dimensional ultrastructural analysis revealed that 86% of the synapses are contacted by an astrocyte (Aten et al., 2022) in a structure known as the “tripartite synapse” (Haydon, 2001), consisting of pre- and postsynaptic elements and an astrocyte. The majority of the synapses contacted by astrocytes are asymmetrical excitatory synapses. The human astrocytes are more than 3–10 times larger than mouse astrocytes, and an astrocyte can ensheath as many as 2 million synapses (Oberheim et al., 2009).

Astrocytes perform a critical function at the synapse (Kimelberg, 2010). Through their K^+^ channels, they remove extracellular K^+^ released during neuronal depolarizations. Their glutamate transporters are used to clear glutamate released from the synaptic cleft (Rothstein et al., 1996), and the transporter level is modulated by neuronal activity such as whisker sensory stimulation (Genoud et al., 2006). The neurovascular unit refers to the strategic cytoarchitecture where an astrocyte surrounds a synapse with a subset of its processes, while the other processes from the same astrocyte surrounds capillaries, facilitating coupling of metabolic function to neuronal activity (Iadecola, 2017). Since astrocytes were the first type of glia to be described, many of the previously observed phenomena attributed to “glia” likely represent not only the function of astrocytes but also the function of OPCs, which came to be recognized as a resident glial population only in the past few decades (Hill et al., 2024).

### 2.2 Oligodendrocyte lineage cells

#### 2.2.1 Mature oligodendrocytes

The primary function of mature oligodendrocytes is to form compact myelin sheaths around axons, allowing for fast, regenerative saltatory conduction of action potentials. The production of myelin and myelinating oligodendrocytes continues through life and is dynamically regulated by neuronal activity (Nishiyama et al., 2021). Oligodendrocytes and myelin provide crucial metabolic support for both neurons and axons to maintain axonal integrity (Griffiths et al., 1998; Lappe-Siefke et al., 2003).

While the model of neurovascular coupling posits that astrocytes are the primary supplier of lactate to neurons during increased neuronal activity fueled by glutamate transporter-mediated glucose utilization leading to lactate release (Pellerin and Magistretti, 1994), oligodendrocytes are also an important supplier of lactate for axons. Oligodendrocytes express monocarboxylate transporter 1 (Lee et al., 2012), which enables lactate uptake from the extracellular space, and its loss in oligodendrocytes causes axonal degeneration without significant demyelination or oligodendrocyte damage (Lee et al., 2012; Philips et al., 2021). Oligodendrocytes increase their intracellular Ca^2+^ in response to axonal activity via the ATP-sensitive inwardly rectifying Kir4.1 potassium channels (Looser et al., 2024). Loss of Kir4.1 in oligodendrocytes leads to failure to increase axonal lactate and glucose consumption during high axonal activity due to a reduction in monocarboxylate transporter 1 and glucose transporter 1. Oligodendrocytes also modulate synapses between the axons they myelinate and the target cells of the axons. Photostimulation of oligodendrocytes in hippocampal CA1 region facilitates synaptic transmission between CA1 neurons and a subset of neurons in the distal subiculum and long-term plasticity at these synapses (Yamazaki et al., 2019).

#### 2.2.2 OPCs

OPCs, identified by the expression of platelet-derived growth factor receptor alpha (PDGFRα), the NG2 proteoglycan, and the oligodendrocyte lineage transcription factor Sox10 (Kuhlbrodt et al., 1998), persist in the mature CNS where they comprise 2%–9% of the cells (Dawson et al., 2003). OPCs arise from different germinal zones in the embryonic CNS and reach their peak density by the end of the first postnatal week (Kessaris et al., 2006; Nishiyama et al., 2016). They are multi-processed, continue to proliferate and self-renew and generate myelinating oligodendrocytes through life. OPCs, like astrocytes, are tiled and occupy non-overlapping domains throughout the CNS while they share territories with astrocytes and microglia. OPC processes are extensively intertwined with astrocyte processes in gray matter (Nishiyama et al., 2002; Hamilton et al., 2010), and they are co-inserted at some nodes of Ranvier (Serwanski et al., 2017). OPC processes are also intimately associated with those of microglia (Sherafat et al., 2021). The uniform distribution of OPCs does not match the distribution of myelin or myelinating OLs, which suggests that OPCs are involved in other functions besides generating myelinating cells (Tomassy et al., 2014).

**FIGURE 2:**
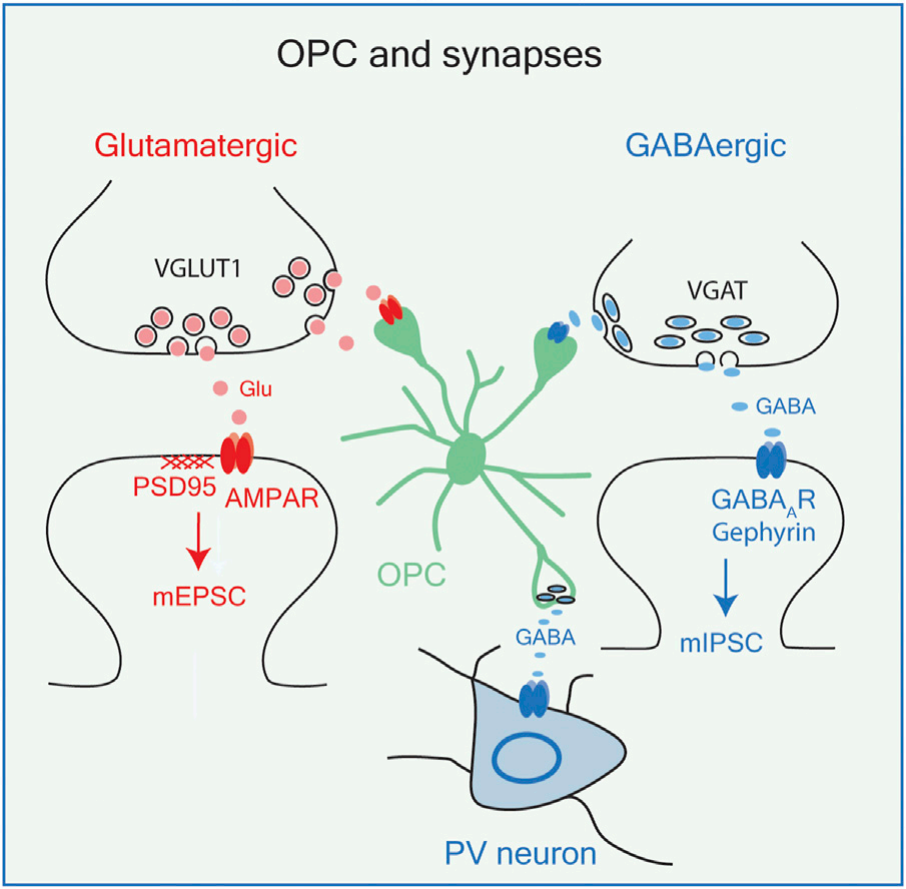
OPCs and synapses. OPCs receive synaptic inputs from glutamatergic neurons (red) and GABAergic neurons (blue), both of which result in membrane depolarizations. OPCs also release GABA on PV (parvalbumin+) interneurons. VGLUT1, vesicular glutamate transporter 1; PSD95. Postsynaptic density protein-95; AMPA R, AMPA receptor; mEPSC, miniature excitatory postsynaptic currents; VGAT, vesicular GABA transporter; GABA_A_R, GABA A receptor; mIPSC, miniature inhibitory postsynaptic currents.

Recent studies have revealed that OPCs carry out some of the functions previously ascribed to astrocytes and microglia, and even neurons. OPCs express voltage-dependent ion channels and glutamate and GABA receptors and receive glutamatergic and GABAergic synaptic inputs from axons (Bergles et al., 2000; Larson et al., 2016) (Figure 2, see also Figure 5A; Supplementary Table S1). These synaptic connections on OPCs are maintained during cell division but are lost as OPCs start to differentiate into myelinating OLs (De Biase et al., 2010; Kukley et al., 2010). Thus, OPCs are uniquely equipped with the cellular mechanism to perceive changes in neuronal activity and neurotransmitter release. OPCs become functionally heterogeneous with respect to their ion channel and neurotransmitter expression as the host animal matures from embryo to adult, and this is reflected in increased regional and age-dependent diversity of their electrophysiological properties (Spitzer et al., 2019). These functional differences are not always accompanied by transcriptomic differences (Marques et al., 2018), suggesting that OPCs exist in different states which may be influenced by an interplay between the environmental factors and their intrinsic ability to respond to external influences, governed by their prior transcriptional history or epigenetic states (Liu et al., 2016; Spitzer et al., 2019).

While the functional consequences of OPC depolarizations induced by neuron-to-OPC synapses have not been fully understood, OPCs express genes known to function at neuronal synapses (see Figure 5; Supplementary Table S1), and new evidence suggests their role at synapses (see below). Furthermore, a recent study showed that photostimulation of OPCs causes vesicular GABA release, leading to phasic and tonic inhibition of nearby interneurons but not pyramidal neurons in the adult hippocampal CA1 region (Zhang et al., 2021) (Figure 2). Although these findings have yet to be replicated, OPCs may not only be recipients of neuronal synapses, but they can form synapses onto inhibitory neurons and engage in bidirectional communication with neurons. It remains to be determined whether these events occur in response to physiological magnitudes of OPC depolarization.

### 2.3 Microglia

Microglia, known as the immune cells and tissue-specific macrophages in the CNS, originate from the primitive yolk sac as primitive macrophages around embryonic day 8.5 (E8.5) in the mouse and begin the colonize the CNS by E9.5. They appear as amoeboid macrophages, self-renew to populate the entire CNS (Ginhoux et al., 2010; Ginhoux and Prinz, 2015), and transform into resting ramified microglia by the end of the second postnatal week (Butovsky et al., 2014). Cell fate and lineage tracing studies combined with bone marrow transplantation and parabiosis experiments have revealed, contrary to the commonly held view, that the resting ramified microglia are replenished by self-renewal in the healthy CNS throughout life and are not replaced by circulating monocytes (Hickey and Kimura, 1988; Ajami et al., 2007; Parkhurst et al., 2013; Ginhoux and Prinz, 2015). Circulating monocytes derived from the bone marrow enter the CNS only under specific non-physiological conditions that involve the loss of blood-brain barrier (BBB) integrity and elevated inflammatory cytokines. However, they are short-lived and do not replace the endogenous microglia. Thus, the resting ramified microglia in the healthy brain are often referred to as “homeostatic microglia,” based on the transcriptomic signatures that distinguish them from the other states (Butovsky et al., 2014; Butovsky and Weiner, 2018; Li et al., 2019; Paolicelli et al., 2022). The classical view that microglia exist in either resting or activated state has been replaced by the view that microglia exist somewhat fluidly along a multidimensional continuum of functional states (Paolicelli et al., 2022).

Microglia are distributed throughout the adult CNS and constitute 5%–12% of the total cells (Lawson et al., 1990), where they are dynamically engaged in homeostatic surveillance function in the absence of pathology. They express a wide variety of ion channels and neurotransmitter receptors (Kettenmann et al., 2011). *In vivo* imaging revealed that microglia in the intact brain have highly dynamic processes that are constantly moving, and they rapidly converge toward the site of elevated extracellular ATP, mimicking ATP released from injured cells (Davalos et al., 2005; Nimmerjahn et al., 2005). Microglia respond rapidly to a small laser-induced lesion of about 15 µm within a minute, and the processes from multiple neighboring microglia converge and contain the lesion within a few minutes in an ATP-dependent manner (Davalos et al., 2005).

During development, microglia phagocytose neurons and glia that are generated in excess and undergo apoptosis in a process called “efferocytosis” (Oppenheim et al., 1991; Morioka et al., 2019). The idea that microglia eliminate synapses was first observed in the facial nucleus after facial nerve injury, where microglia were seen to be “stripping” degenerated axon terminals (Blinzinger and Kreutzberg, 1968). More recent evidence suggests that microglia not only strip synapses after injury but are actively involved in regulating synapses and neuronal excitability in physiological states. In the adult visual cortex during normal visual experience, microglial processes contact axon terminals, postsynaptic dendritic spines, and profiles of astrocytes in a dynamic process lasting from 5 to 50 min, leading to changes in the size or elimination of the contacted synapses (Wake et al., 2009; Tremblay et al., 2010), as discussed in detail below.

## 3 Glia in synapse formation

The CNS is wired in different phases during development. In the developing mouse neocortex, excitatory neurons are first specified in the pallial germinal zone and migrate radially to their final location as they develop their axons and dendrites. This is followed by a gliogenesis phase that generates astrocytes and later oligodendrocyte lineage cells postnatally. Another cohort of earlier-born OPCs arise from the ventral germinal zones and begin their tangential migration together with interneurons that continues until after birth (Anderson et al., 1997; Kessaris et al., 2006; Orduz et al., 2019).

During the early postnatal phase of synapse formation, multiple immature synapses are formed on target neurons, which subsequently undergo activity-dependent refinement during the critical period of synaptic plasticity. While astrocytes were considered to play a major role in synaptogenesis, recent studies have revealed that microglia also positively regulate synapse formation. We do not yet know whether and how OPCs affect neuronal synapse formation, but given that they receive synaptic inputs from neurons, it is reasonable to speculate that they have a role in regulating neuronal synapse formation.

### 3.1 Astrocyte-derived factors promote synapse formation

#### 3.1.1 Astrocytes and excitatory synapse formation

Once axons find their way to the target area, they make contacts with their targets, which then undergo structural and molecular specialization to become a functioning synapse. Early *in vitro* studies revealed that “glial cells” from different CNS regions, which were likely to have been astrocytes, induce morphological differentiation of mesencephalic dopaminergic neurons in a region-specific manner (Denis-Donini et al., 1984). Subsequently, as we began to better understand the specific glial cell subtypes that exist in the CNS, experiments were conducted to specifically address the role of astrocytes in synaptogenesis. Purified retinal ganglion cells from early postnatal rodent retina form synapses in culture. Media conditioned by astrocytes from the tectum, the target of retinal ganglion cells, promote the formation of functional synapses (Pfrieger and Barres, 1997) shown by increased frequency and amplitude of spontaneous excitatory postsynaptic currents (EPSCs) recorded from retinal ganglion cells. Curiously, the authors reported that both astrocytes and oligodendrocytes from the tectum increase the frequency of EPSCs in retinal ganglion cells in a non-contact-mediated manner, whereas microglia have no effect. Subsequent efforts focused on the astrocyte-derived synaptogenic factors.

**FIGURE 3:**
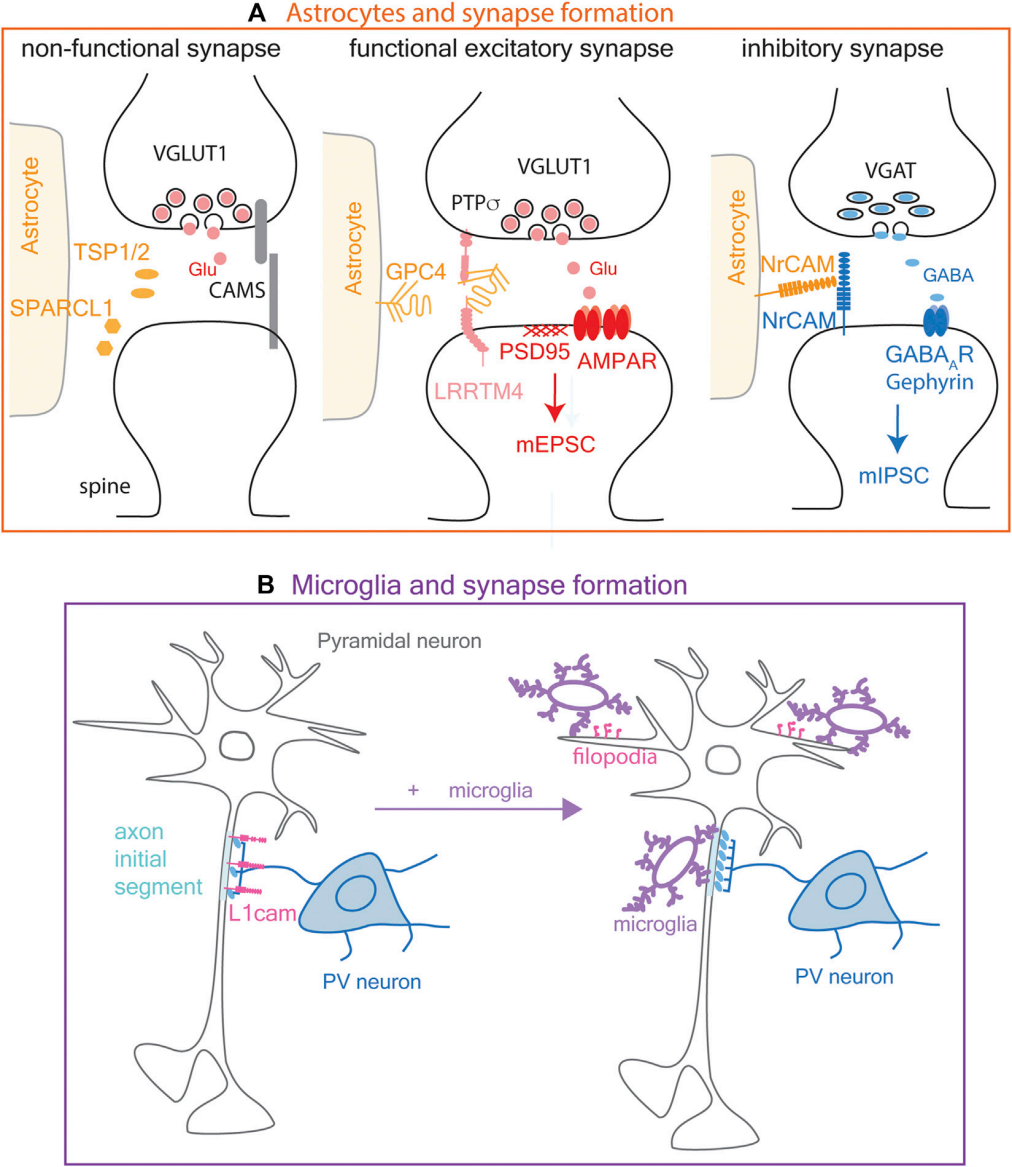
Modulation of synapse formation by astrocytes and microglia. **(A)**. Astrocyte modulation of synapse formation. **Left:** Astrocytes modulate excitatory synapse formation by secreting extracellular proteins TSP1/2 (thrombospondin 1 and 2) and SPARCL1 (secreted protein, acidic and rich in cysteine-like 1, also known as Hevin), which promote morphological synapse differentiation but not functional maturation. **Middle:** Glypican 4 (GPC4) shed from astrocyte surface interacts with PTPσ (protein tyrosine phosphatase sigma) and LRRTM4 (leucine-rich repeat transmembrane neuronal proteins 4) on pre-and postsynaptic membranes, respectively, to promote functional synapse maturation characterized by clustering of AMPA receptors and mEPSC (miniature excitatory postsynaptic current) generation. **Right:** Astrocytes modulate inhibitory synapse formation partly via NrCAM (neural cell adhesion molecule) expressed on their surface, through its homophilic adhesion with NrCAM on the postsynaptic membrane. **(B)**. Microglia promote inhibitory synapse formation by the axon terminals of PV (parvalbumin) neuron at the axon initial segment of a pyramidal neuron where L1cam adhesion molecules are present on the cell surface (left). Microglia also induce filopodia formation (pink) from dendrites at excitatory neuronal synapses (right).

Extracellular matrix proteins thrombospondin-1 and 2 and SPARCL1 (secreted protein, acidic and rich in cysteine-like 1, also known as Hevin) are secreted from astrocytes and promote the morphological differentiation of asymmetric synapses between retinal ganglion cells and their targets in the tectum (Figure 3A, left; Christopherson et al., 2005; Kucukdereli et al., 2011). The effect of thrombospondins is mediated by α2δ-1, which is also known as the receptor for the anti-epileptic drug gabapentin, originally designed as a GABA analog (Eroglu et al., 2009; Risher et al., 2018). However, synapses formed in response to thrombospondins and SPARCL1 are postsynaptically silent and do not recruit sufficient AMPA receptors on the postsynaptic membrane to generate EPSCs.

Fully functional synapses are formed in response to another astrocyte-derived protein, glypican-4 (Figure 3A, middle), which is a glycosylphosphatidylinositol-anchored heparan sulfate proteoglycan. Glypican-4 on astrocytes is cleaved to generate soluble glypican-4, which stabilizes and increases the surface expression of the GluR1 AMPA receptor subunit (Allen et al., 2012). Glypican-4 binds to LRRTM4 (leucine-rich repeat transmembrane neuronal proteins 4) on hippocampal neurons and layer 2/3 cortical neurons (de Wit et al., 2013). Furthermore, protein tyrosine phosphatase sigma (PTPσ) binds to the cleaved glypican-4-LRRTM4 complex and promotes LRRTM4-dependent formation and transmission of excitatory synapses (Ko et al., 2015). OPCs also express *Lrrtm4* and *Ptprs* transcripts (see below in Figure 5; Marques et al., 2018; Zhang et al., 2014), but the role of the OPC-derived proteins has not been elucidated.

#### 3.1.2 Astrocytes and inhibitory synapse formation

Astrocytes not only promote the formation of excitatory synapses, but they modulate GABAergic synapses through homophilic cell adhesion between neuronal cell adhesion molecule (NrCAM) on astrocyte membrane and neuronal presynaptic membrane (Figure 3A, right). Deletion of astrocyte NrCAM led to increased distance between astrocyte membrane and VGAT (vesicular GABA transporter)+ inhibitory presynaptic terminal but not VGlut1 (vesicular glutamate transporter)+ excitatory presynaptic terminal (Takano et al., 2020). Furthermore, deletion of astrocyte NrCAM led to a significant decrease in the frequency and amplitude of miniature inhibitory postsynaptic currents (mIPSCs), further supporting that GABAergic synapses are modulated by astrocyte NrCAM. Curiously, while astrocytes express a higher level of *Nrcam* transcript than other cell types, mouse OPCs also express a significant level of *Nrcam* mRNA, even more than neurons (see below in Supplementary Table S1; Figure 5A; Marques et al., 2018; Zhang et al., 2014).

### 3.2 Microglia and synapse formation

#### 3.2.1 Microglia and excitatory synapse formation

While microglia are most extensively studied in the context of synapse remodeling (see section 4 below), there is evidence that microglia also contribute to synapse formation. Live imaging in layer 2/3 of the developing somatosensory cortex shows that during a limited developmental window between postnatal day 8 and 10 (P8-10) in mice, microglial contacts cause a rise in Ca^2+^ in the contacted dendrites followed by filopodia extension (Figure 3B), which presumably develop into functional excitatory synapses. Ablation of microglia reduces filopodia formation. Furthermore, photostimulation of caged glutamate across the cortical layers results in reduced EPSCs in layer 2/3 neurons in microglia-ablated cortex, indicating that microglia play a critical role in the development of dendritic filopodia into excitatory synapses (Parkhurst et al., 2013; Miyamoto et al., 2016). Live imaging revealed that genetic ablation of microglia at P19 or P30 reduces the formation of new spines in the motor cortex (Parkhurst et al., 2013). The timing of microglial ablation in this study was designed to specifically target resident microglia and not the short-lived blood monocyte-derived macrophages which also express CX3CR1. These findings suggest that homeostatic microglia are involved in developmental excitatory synapse formation independently of synapse elimination.

#### 3.2.2 Microglia and inhibitory synapse formation

Microglia also promote GABAergic inhibitory synapse formation at the axon initial segment of pyramidal neurons in the mouse somatosensory cortex during the first three postnatal weeks (Figure 3B), when microglial association with the axon initial segment is the highest (Gallo et al., 2022). The synaptogenic effect of microglia is dependent on microglial GABA_B_ receptor, which is involved in recruiting microglial processes to the axon initial segment (Gallo et al., 2022). The initial contact between axon terminals of the inhibitory neuron and its target axon initial segment of the pyramidal cell is dependent on axonal L1CAM but does not require microglia (Tai et al., 2019). Microglia are needed in the subsequent step of maturation of these contacts into functional synapses (Gallo et al., 2022). Thus, in addition to the well characterized roles of astrocytes in synaptogenesis, emerging evidence suggests that microglia are also involved in the formation of both excitatory and inhibitory synapses.

## 4 Glia in synapse maturation and refinement

During the initial synaptogenesis phase described above, synapses are formed in excess. Subsequently, synaptic remodeling eliminates weak synapses and strengthens active synapses. Here we describe examples of glial involvement in some of the well studied paradigms of synapse refinement and plasticity.

### 4.1 The role of microglia in synapse refinement

#### 4.1.1 Early retinogeniculate activity-dependent synapse refinement by C1q-CR3 signaling

**FIGURE 4:**
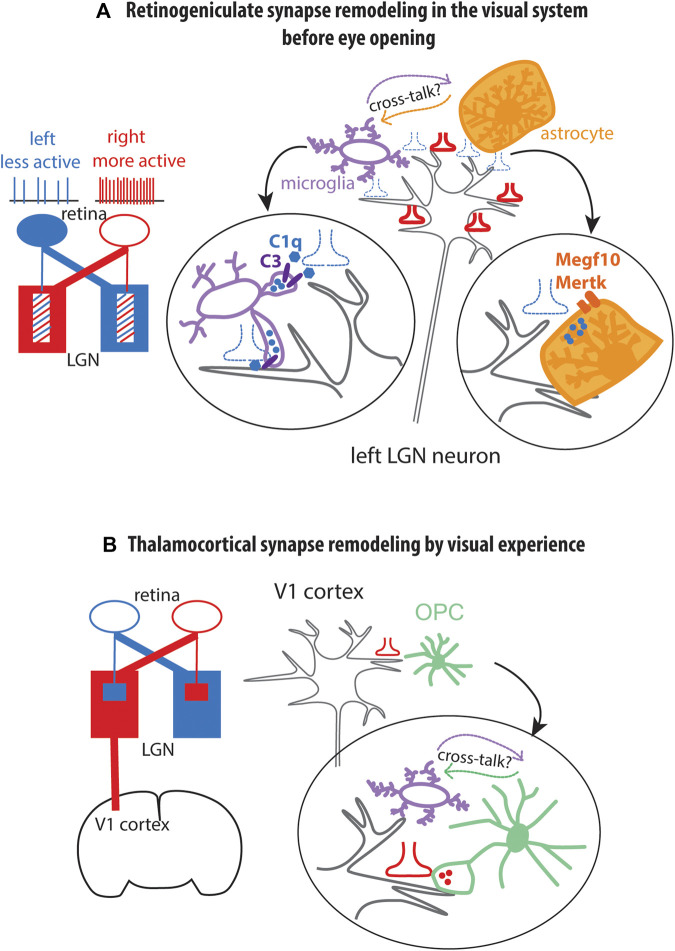
Modulation of synapse remodeling by glia **(A)**. Early postnatal synapse remodeling at the retinogeniculate synapse prior to eye opening. Schematic in blue and red indicate initial bilateral innervation of lateral geniculate nucleus by retinal ganglion cell axons. During this period, weak synaptic inputs (blue, dotted) are engulfed by microglia through C1q-C3 interaction and by astrocytes by the phagocytosis receptors Megf10 (Multiple epidermal growth factor-like domains protein 10) and Mertk (Tyrosine-protein kinase Mer). The respective roles of microglia and astrocytes in engulfing synapses and their cross-talk are not known. Blue and red symbols indicate presynaptic terminals. Blue circles represent presynaptic terminals found inside microglia or astrocytes suggesting engulfment. LGN lateral geniculate nucleus. **(B)**. Synapse remodeling by microglia and OPC (oligodendrocyte precursor cell) in the primary visual cortex (V1 cortex) during visual experience after eye opening at P12. Red presynaptic terminal symbols represent lateral geniculate axons. Red circles inside OPC process represent engulfed presynaptic terminal. Microglia are needed for OPCs to engulf synapses, but the nature of the cross-talk remains unknown.

The early phase of retinogeniculate synapse refinement peaks around P5 and declines by P9, prior to eye opening at P12-13. Initially, inputs from the two eyes target overlapping regions in the lateral geniculate nucleus. Gradually, the mistargeted inputs are eliminated so that retinal ganglion cell axons from each eye segregate into specific layers in the lateral geniculate nucleus (Figure 4A). This type of synaptic refinement is dependent on spontaneous retinal ganglion cell activity (Katz and Shatz, 1996; Hong and Chen, 2011; Schafer et al., 2012). During this phase of synaptic remodeling, microglia engulf weaker presynaptic terminals (Figure 4A). The engulfment of weak synapses by microglia occurs by opsonization of weak synapses with C1q, a large secreted protein and initiator of the classical complement pathway, which provides an “eat me” signal for microglia (Stevens et al., 2007), acting on the complement receptor CR3 expressed on microglia. By contrast, active synapses carry CD47, which binds to its receptor SIRPα expressed on microglia and functions as a “do not eat me” signal (Lehrman et al., 2018).

#### 4.1.2 Visual experience-dependent synapse refinement and paracrine signaling

After eye-opening, retinogeniculate synapses and thalamocortical synapses undergo activity-dependent refinement during the critical period of synapse plasticity between P20 and P30 (Hong and Chen, 2011). Dark-rearing between P20 and P27 increases microglial contacts with synapses in the primary visual cortex, resulting in shrinkage of synapses (Tremblay et al., 2010). At the retinogeniculate synapses in the lateral geniculate nucleus, synapse elimination during sensory experience-dependent plasticity period can occur in the absence of engulfment of synapses by microglia, by a mechanism that involves paracrine signaling between TWEAK (TNF ligand superfamily member 12) secreted from microglia repressing the synapse-strengthening effects of its receptor Fn14 encoded by *Tnfrsf12a* (tumor necrosis factor (TNF) receptor superfamily 12a) expressed on the postsynaptic spine (Cheadle et al., 2020).

#### 4.1.3 Synapse refinement in the whisker barrel cortex by CX3CL1-CX3CR1 signaling

The thalamocortical projection to the whisker barrel cortex is often used to study whisker sensory activity-dependent changes. Sensory input from individual whiskers is topographically mapped to a specific barrel in layer IV of the primary somatosensory cortex. Removal of a whisker results in loss of input to the corresponding barrel in the contralateral barrel cortex.

In the developing barrel cortex, clustered fractalkine (CX3CL1) expression in the barrels is detected around P5, shortly followed by the invasion of microglia from the barrel septum into the barrel hollow where the thalamocortical axons from each whisker input are clustered (Hoshiko et al., 2012), and its receptor CX3CR1 is needed for the postsynaptic maturation of glutamate receptors. Unilateral whisker removal at P4 results in loss of thalamocortical synapses in the contralateral barrel cortex due to microglial engulfment of the presynaptic terminals (Gunner et al., 2019). In whisker-deprived mice, Adam10 (Disintegrin and metalloproteinase domain-containing protein 10) cleaves CX3CL1, and soluble CX3CL1 interacts with its receptor CX3CR1 on microglia to initiate synapse engulfment. CX3CR1-dependent synapse engulfment affects primarily the presynaptic terminals and not the postsynaptic portions. CX3CL1-CX3CR1 signaling is also critical in synapse elimination by microglia in the hippocampus (Paolicelli et al., 2011).

#### 4.1.4 Inhibitory synapse refinement by microglia

Microglia not only remodel excitatory synapses but are also involved in remodeling inhibitory synapses. In the postnatal somatosensory cortex, a subset of microglia that express GABA_B_ receptors 1 and 2 and are GABA-receptive are preferentially associated with parvalbumin+ (PV+) inhibitory boutons around the soma of principal neurons (Favuzzi et al., 2021). When *Gabbr1* is deleted in microglia, the number of PV+ presynaptic boutons around neuronal soma increases, and this is accompanied by increased mIPSC frequency, leading to altered excitation/inhibition balance affecting mouse behavior. Indirect evidence suggests that the remodeling of inhibitory synapses by GABA-receptive microglia is mediated by synaptic pruning.

#### 4.1.5 Refinement of climbing fiber to Purkinje cell synapses requires C1QL1 and microglia

A similar refinement occurs between climbing fibers and Purkinje synapses in the developing cerebellar cortex. Initially Purkinje cells receive multiple synapses from climbing fibers during the first postnatal week. By the beginning of the second postnatal week, excess climbing fiber synapses are eliminated so that ultimately synapses from one climbing fiber innervate each Purkinje cell, and the winner climbing fiber translocates to form multiple synapses along the dendritic arbor of the target Purkinje cell by the end of the third postnatal week (Hashimoto and Kano, 2013). There are extracellular proteins that belong to the C1q-like family (C1QL1-4), which are structurally related to C1q. C1QL1 is expressed by the presynaptic terminals of climbing fibers (Kakegawa et al., 2015). When *C1ql1* is deleted from neurons of the inferior olivary nucleus, innervations from multiple on each Purkinje cells persist, and the subsequent translocation of the “winner” climbing fiber synaptic terminals along the dendrites of the target Purkinje cell is impaired (Kakegawa et al., 2015). Microglia are involved in this process, which is mediated by modulation of GABAergic inhibition rather than synapse engulfment (Nakayama et al., 2018).

### 4.2 The role of astrocytes in synapse elimination and refinement

Astrocytes also contribute to synapse elimination. Transcriptomic analysis revealed that astrocytes express *Megf10* and *Mertk* genes encoding phagocytic receptors Multiple epidermal growth factor-like domains protein 10 and Tyrosine-protein kinase Mer, respectively (Chung et al., 2013). In the developing retinogeniculate system, *Megf10* and *Mertk* genes are expressed in early postnatal lateral geniculate nucleus where retinal ganglion cell projections terminate. Presynaptic terminals of retinal ganglion cell neurons are found in the endosomal/lysosomal compartment of astrocytes, suggesting that they are engulfed by astrocytes (Figure 4A). In *Megf10* ^−/−^ or *Metk* ^−/−^ mice, astrocytes fail to engulf weaker projections, leading to a failure of retinal ganglion cell projections into separate eye-specific layers in the lateral geniculate nucleus. Astrocyte-mediated synapse elimination also occurs in the adult hippocampus in an activity-dependent manner (Lee et al., 2021). Additionally, Bergmann glia in the cerebellar cortex engulf parallel fiber to Purkinje cell synapses during motor learning (Morizawa et al., 2022). However, the cross-talk between astrocytes and microglia in synapse engulfment has not yet been explored (Figure 4A).

### 4.3 The role of OPCs in synapse refinement

Recent findings suggest that OPCs are engaged in the refinement of axon branching (Xiao et al., 2022) and synapses (Auguste et al., 2022; Buchanan et al., 2022), independently of their role in generating myelinating cells. In the visual system, OPCs engulf synapses during remodeling of thalamocortical inputs from the lateral geniculate nucleus to the primary visual cortex in mice (Auguste et al., 2022). GFP-labeled thalamocortical axons are detected inside the OPCs in the visual cortex at all stages of synapse remodeling (Figure 4B). VGLUT2+ thalamocortical axon terminals in the OPC are colocalized with the phagolysosomal marker LAMP2, and the fluorescence of GFP-labeled thalamocortical terminals is quenched inside OPCs, further providing evidence that endocytosed axon terminals are trafficked to the acidic phagolysosomal compartment in OPCs. *In vivo* imaging revealed that OPCs in the adult visual cortex contact multiple thalamocortical axons and engulf smaller inputs compared to those that are not engulfed. The ability to engulf thalamocortical terminals is unique to OPCs and is not seen in mature oligodendrocytes. Intriguingly, engulfment of thalamocortical material by OPCs is more extensive than engulfment by microglia at all time points examined but is reduced when microglia are pharmacologically ablated (Auguste et al., 2022; Figure 4B).

Another study (Buchanan et al., 2022) conducted a detailed 3D ultrastructural analysis of OPCs in the adult mouse visual cortex by serial transmission electron microscopy. At P36 OPC processes contain phagosomes and phagolysosomes, some of which have clear vesicles that resemble synaptic vesicles, suggesting that OPCs engulf synapses. Fewer phagolysosomes are detected in microglia which share the neuropil domain with OPCs. Furthermore, OPCs in the adult cortex express transcripts encoding proteins involved in phagocytosis (Yao et al., 2021; Buchanan et al., 2022). *In vitro* studies suggest that OPCs and microglia phagocytose at different rates. When rat OPCs are confronted with debris, they take up the debris after 24 h, whereas microglia phagocytose the debris after 2 h. Transcriptomic analysis revealed that genes related to oligodendrocyte differentiation and myelination are downregulated in phagocytosing OPCs while immediate early genes such as *Egr1/2*, *Junb*, and *Fos* are upregulated (Hamanaka et al., 2023).

OPCs not only engulf synaptic terminals but they also contain profiles of excitatory and inhibitory axons, indicative of their role in axonal remodeling (Buchanan et al., 2022). This is consistent with the findings in the zebrafish optic tectum where OPCs that do not readily differentiate into myelinating oligodendrocytes prune retinotectal axons and play a critical role in shaping the neural circuit affecting visual behavior (Xiao et al., 2022). Collectively these new observations suggest that OPCs not only passively receive synaptic inputs from neurons but are actively involved in neural circuit refinement.

## 5 Glial cross-talk in network function

### 5.1 Detection of neuronal activity by glia

If glia were involved in synapse elimination during the critical period of synaptic plasticity, there must be a mechanism by which they detect neuronal activity and discriminate between strong and weak synapses. Microglia detect weak synapses marked for elimination by a molecular tag described above. They also respond to changes in neuronal activity by dynamically altering their cellular behavior. For example, homeostatic microglia make contacts with synapses approximately once every hour, and this frequency decreases when neuronal activity is blocked (Wake et al., 2009). In cortical slices, electrical stimulation of neurons causes axonal swelling, and within 10–15 min, microglia wrap around the swollen axons via a mechanism that involves volume-activated anion channels. When these channels are pharmacologically blocked, the microglia no longer wrap around the stimulated axon, and the neuronal membrane continues to depolarize irreversibly, leading to excitotoxic death (Kato et al., 2016). Microglia detect an increase in neuronal activity via neuronally derived ATP that acts on microglial P2Y12 receptors and negatively regulates neuronal firing (Badimon et al., 2020). Microglial processes with clustered P2Y12 receptors make specialized contacts at subdomains of the neuronal somatic membrane with clustered Kv2.1, which represent sites of high ATP production from nearby mitochondria (Cserep et al., 2020). Recruitment of microglial processes to these sites is dependent on P2Y12 receptor activation via ATP released from activated neurons and converted into ADP. Furthermore, in an ischemic lesion, P2Y12-dependent microglial coverage of neuronal membranes at sites of high ATP production protects neurons from calcium overload and limits tissue damage. Collectively, these findings suggest an ability of microglia to detect increased neuronal activity and impart a neuroprotective signal through the neuronal subdomains they contact. These types of neuron-microglial interactions appear to occur at non-synaptic sites.

The ability of OPCs to receive synaptic inputs suggests that OPCs also detect neuronal activity (Hill and Nishiyama, 2014; Nishiyama et al., 2021; Xiao and Czopka, 2023). Indeed OPCs undergo changes in response to increased neuronal activity. For example, OPCs proliferate in response to photostimulation of the motor cortex (Gibson et al., 2014). Pharmacogenetic or optogenetic stimulation of callosal fibers promotes remyelination after chronic or acute demyelinating injury (Mitew et al., 2018; Ortiz et al., 2019). In zebrafish, some synaptic sites that are formed on OPCs serve as hotspots for sites of future myelination (Li et al., 2024). However, we do not yet fully understand the nature of signals that active neurons impart on OPCs to initiate a cascade of signaling that ultimately leads to altered cellular behavior. In a new study mature oligodendrocytes were shown to increase their intracellular Ca^2+^ in response to increased axonal activity and a subsequent rise in extracellular K^+^ in the optic nerve, which is mediated by oligodendroglial Kir4.1 potassium channels and NCX sodium calcium exchanger (Looser et al., 2024). The oligodendrocyte response to nerve stimulation is accompanied by increased glucose uptake by a Kir4.1-dependent mechanism, leading to lactate supply in axons. Whether these mechanisms also operate in OPCs to detect axonal activity outside synaptic sites remains to be tested.

### 5.2 OPCs express genes encoding proteins known to function at synapses

**FIGURE 5:**
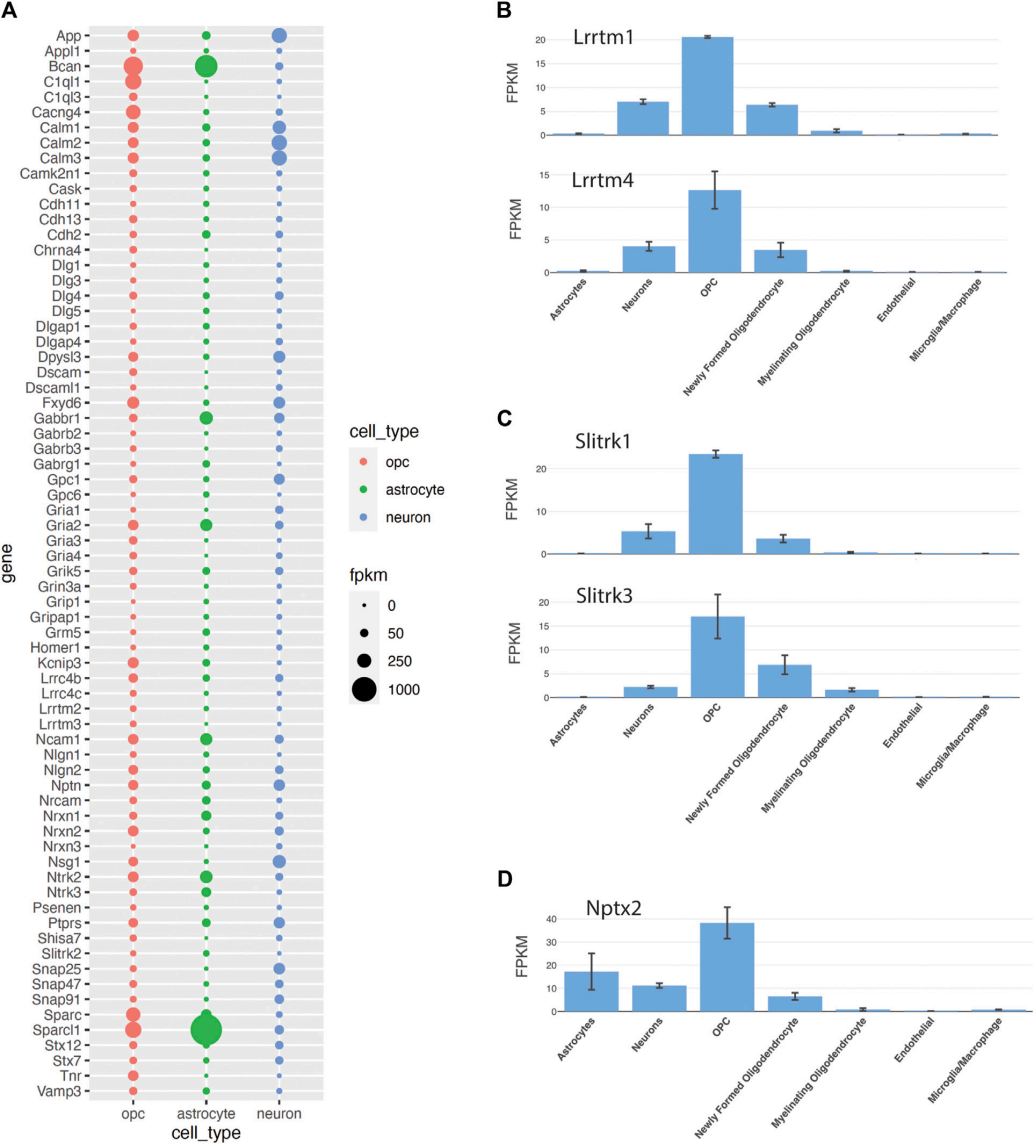
Expression of synaptic genes in OPCs **(A)**. Fpkm values of 70 selected genes out of 564 genes that are in the “synapse” GO term and are expressed in OPCs in bulk- and single-cell-RNA-sequencing data (Zhang et al., 2014; Marques et al., 2018). The sizes of the bubbles are the average FPKM values of OPC1 and OPC2, astrocyte 1 and astrocyte 2, and neuron 1 and neuron 2 from the Zhang database. (**B–D)**. Genes that were not among the 564 genes identified from the “synapse” GO term but also implicated in synapses, including Lrrtm **(B)** and Slitrk **(C)** family of genes and Nptx2 **(D)**, a known neuronal derived synaptic modulator expressed by OPCs, according to the Zhang et al., 2014 database.

According to the available transcriptomic databases, OPCs express transcripts encoding proteins implicated in synapse development and maintenance that have been primarily ascribed to neurons and astrocytes. To examine the expression of synapse-related genes in OPCs, we took the intersection of 1743 genes in the gene ontology term “synapse” (GP 0045202) and genes shown to be expressed in OPCs in a bulk RNA-seq dataset from P17 mouse cortex (Zhang et al., 2014) and in a single-cell RNA-seq dataset from P20-60 forebrain regions (Marques et al., 2018), which identified 564 common genes (Supplementary Table S1). The abundance of 70 of these genes in OPCs, astrocytes, and neurons here is shown in Figure 5A. The list includes genes encoding receptors for neurotransmitters, such as glutamate, GABA, and acetylcholine and genes that regulate trafficking and stability of these receptors (*Dlg1,3–5; Dlgap1,4; Grip1, Gripap1, Homer1*). In addition to the core neuronal synapse genes, OPCs express genes encoding proteins that are known to be secreted from astrocytes and regulate synaptogenesis, such as *Sparc, Sparcl1, Bcan, Ncam1, Nrcam, Gpc1* and *6*. However, it is not yet known whether OPCs secrete these gene products at different sets of synapses to those affected by astrocyte-derived proteins or whether the OPC-derived proteins augment or diminish the function of those from astrocytes at the same synapse. Furthermore, OPCs express genes encoding integral membrane synaptic adhesion proteins including *Cdh2, 11, 13; Dscam, Dscaml1, Lrrc4b, c; Lrrtm1-4*; *Sltrk1-3*, *Nrxn1-3, Nlgn1-2,* and *Ptprs* (Figure 5B,C). It would be interesting to determine whether the same set of synaptic adhesion proteins are used in functionally coupled neuron-neuron and neuron-OPC synapses and how the cellular sources affect their function.

*C1ql1* is one of the highest-expressing synaptic genes in OPCs (Figure 5A; Zeisel et al., 2018). New studies suggest that *C1ql1* is expressed in OPCs in the neocortex (Altunay et al., 2024; Moghimyfiroozabad et al., 2024). While neuronally derived C1QL1 plays a critical role in remodeling synapses between climbing fibers and cerebellar Purkinje cells (see above, Section 4.1.5), the role of OPC-derived C1QL1 at the synapse in the forebrain is not known. Since C1QL1 is a large secreted extracellular protein, it could potentially affect both neuron-OPC synapses and adjacent neuron-neuron synapses.

### 5.3 Cross-talk between microglia and OPCs

As described above in Section 4, both microglia and OPCs are involved in synaptic refinement (Stevens et al., 2007; Schafer et al., 2012; Auguste et al., 2022). Microglia remodel synapses via engulfment of weak synapses by C1q-CR or CX3CL1-CX3CR1 signaling or by a paracrine mechanism that involves secretion of TWEAK modulating the function of Fn14 on the postsynaptic membrane. OPCs engulf more synapses in the developing visual cortex than microglia, but the ability of OPCs to engulf synapses is dependent on microglia (Cheadle et al., 2018). However, the nature of the communication between microglia and OPCs during visual experience-dependent synaptic refinement is unknown (Figure 4B). Interestingly, OPCs also secrete TWEAK by a mechanism that requires GABA_B_R1 receptor (Fang et al., 2022). How the two sources of TWEAK affect signaling through Fn14 at the synaptic spines remains unknown.

In the early postnatal white matter tracts such as the corpus callosum, there is a close spatial and functional relation between OPCs and amoeboid microglia, also known as axon tract-associated microglia (ATM) or early postnatal proliferative region-associated microglia (PAM) with a distinct transcriptomic signature (Hammond et al., 2019; Li et al., 2019). One study shows that these microglia phagocytose OPCs in a process that requires CX3CR1 on microglia (Nemes-Baran et al., 2020). On the other hand, we have shown that ATM/PAM microglia in the corpus callosum at this developmental stage express neuropilin-1, which phosphorylates PDGFRα on adjacent OPCs *in trans* and promotes OPC proliferation (Sherafat et al., 2021). These seemingly contradictory observations in the white matter could represent different ends of a spectrum of how microglia regulate OPC density and hence oligodendrocyte and myelin density (Sherafat et al., 2022). Since synaptic transmission also occurs in the white matter between callosal axonal profiles and OPCs (Ziskin et al., 2007; Kukley et al., 2010) at structures previously described as axoglial synapses (Mugnaini and Walberg, 1964; Palacios-Pru et al., 1983; Peters et al., 1991), OPCs could be gauging the level neuronal activity and relaying the information to microglia to remodel developing axons (Innocenti and Clarke, 1983; Elberger, 1993; Kadhim et al., 1993). While OPCs have been shown to remodel retinotectal axonal arbor in zebrafish as described above (Xiao et al., 2022), it is not known whether this process is dependent on microglia. The complexity and reciprocity of microglia-OPC cross-talk is further illustrated by the observation that loss of OPCs perturbs the homeostatic state of microglia in the cerebral cortex leading to downregulation of genes encoding functionally important proteins such as CX3CR1 and P2Y12 and TGFβ (Liu and Aguzzi, 2020).

## 6 Glial cross-talk in pathological states

### 6.1 Excessive synapse elimination in schizophrenia by microglia

Synapse elimination has been implicated in several neuropsychiatric disorders including schizophrenia. It has been known for almost half a century that the number of synapses in the cortex of the human frontal lobe increases significantly during the first 5 years of life, after which it decreases through adolescence and plateaus during adult life until it starts to decline after 60 years of age (Huttenlocher, 1979). Clinical imaging studies revealed loss of gray matter volume, but no overt neuronal loss had been detected in pathological studies. More sophisticated morphological techniques revealed a significant decrease in synapse density in the prefrontal cortex of patients with schizophrenia that begins in adolescence, coinciding with onset of symptoms, and remains low in the adult (Penzes et al., 2011; Glausier and Lewis, 2013). By contrast, autism spectrum diseases have increased number of spines starting in childhood through adolescence and adulthood (Neniskyte and Gross, 2017).

Recent genetic studies have identified variations in *C4A* and *C4B* genes encoding complement component 4a and 4b within the major histocompatibility locus on chromosome 6 as a risk factor for schizophrenia (Sekar et al., 2016). Alleles that generate higher levels of C4A are associated with greater risk. C4 protein is found in neurons in the human hippocampus and is colocalized with the synaptic proteins PSD-95 and VGLUT1 and 2. In the absence of C4, deposition of C3, the target of C4, is reduced at synapses, and retinogeniculate synaptic refinement is compromised.

To model neuron-microglial interaction in schizophrenia, one study examined the ability of microglia from control or schizophrenia patient-derived induced pluripotent stem cells (iPSCs) (Sellgren et al., 2019). When synaptosomes from schizophrenia patients are presented to microglia derived from patients’ iPSCs, synapse engulfment is most prominently observed between patient-derived synaptosomes and patient-derived microglia. This is further supported in a more recent study using cocultures of neurons and microglia derived from iPSCs from control and schizophrenia subjects (Breitmeyer et al., 2023). Microglia from schizophrenia iPSCs exhibit a higher level of inflammatory activity and greater ability to engulf presynaptic terminals. The increased synapse engulfment by schizophrenia patient-derived microglia in both studies could be reversed by the anti-inflammatory drug minocycline (Sellgren et al., 2019; Breitmeyer et al., 2023).

### 6.2 Reduction of neuronal synapses in schizophrenia by hyperactive Wnt signal in OPCs

In the cortex and hippocampus of patients with schizophrenia, OPCs exhibit greater morphological complexity (Yu et al., 2022). This phenotype is reproduced in a mouse model of schizophrenia that expresses a mutant form of the *Disc1* (Disrupted in schizophrenia homolog 1) gene specifically in OPCs and their progeny. These mice exhibit schizophrenia-like behaviors, have reduced excitatory synapses in hippocampal CA1 region, and exhibit reduced frequency of EPSCs. The hypertrophic OPCs in the mutant mice make more contacts with neurons that have reduced synapses at an age when there is no myelin defect. Furthermore, the mutant OPCs have hyperactive Wnt/β-catenin signaling, which triggers an increase in the expression of the secretory protein Wif1 (Wnt inhibitory factor 1) in OPCs. Reducing OPC-derived Wif1 rescues the synapse defects in mice expressing mutant *Disc1* in OPCs. Thus, OPCs may be directly affecting excitatory neuronal synapse numbers in schizophrenia. Whether there is a functional cross-talk between OPCs and microglia in synapse reduction in schizophrenia is not known.

### 6.3 Synapse elimination in dementia disorders

Some of the molecules involved in synapse development are also altered in dementia disorders. For example, neuronal pentraxin 2 (NPTX2, originally named Narp) is a member of the Pentraxin family with shared structure (Hsu and Perin, 1995; Tsui et al., 1996). Its expression increases during the postnatal 3 weeks, coincident with synapse maturation (Tsui et al., 1996). *Nptx2* expression is upregulated in the hippocampus within an hour after high frequency synaptic stimulation that induces long-term potentiation (LTP). NPTX2 is a secreted protein that is found at excitatory synapses on interneurons and causes clustering of GluR4 AMPA receptor subunits on fast-spiking PV+ interneurons (Pelkey et al., 2015). Neuronal pentraxin-1 and 3 (NPTX1 and 3) can also mediate AMPA receptor recruitment, leading to increased mEPSC, and this process can be negatively regulated by thrombospondins (Bjartmar et al., 2006).

NPTX2 and other family members of NPTX proteins bind to C1q and inhibit the activation of the classical complement pathway (Zhou et al., 2023). In *Nptx2* knockout hippocampus, there is increased C1q-decorated excitatory synapses at PV+ interneurons and their increased localization in lysosomes. When NPTX2 is overexpressed in neurons in the Tau P301S model of frontotemporal dementia (FTD) and Alzheimer’s Disease (AD), the level of the complement C4b and synapse engulfment decreases, leading to increased number of excitatory synapses on parvalbumin interneurons. NPTX2 levels are lower in the CSF of FTD patients (Zhou et al., 2023), and reduced CSF NPTX2 levels can be used as a predictor of cognitive impairment in AD (Soldan et al., 2023).

*Nptx2* transcript is also detected in OPCs (Figure 5D; Marques et al., 2018; Zhang et al., 2014), and NPTX2 protein is detected in cultured OPCs from P9 mouse brain but disappears as they differentiate into OLs (Sakry et al., 2015). While the specific role of OPC-derived NPTX2 in synaptic function remains to be tested, it may play a modulatory role in neuronal synapse formation in physiological and pathological conditions.

### 6.4 Synapses and network function in glioma

OPCs are considered to be the cell of origin of many gliomas (Liu et al., 2011; Galvao et al., 2014). Neuron-OPCs synapses are retained in glioma (Venkatesh et al., 2019), and neuronal activity drives glioma proliferation through a paracrine mediator, such as neuroligin-3 from neurons (Venkatesh et al., 2015; Mancusi and Monje, 2023). Recent studies indicate that malignant glioma cells can enhance network connectivity in the affected cortex (Krishna et al., 2023), and the extent of network connectivity is associated with worse clinical outcomes. Gliomas exhibiting high functional connectivity express thrombospondin-1, which is an astrocyte-derived synaptogenesis factor as described above. Treatment of mice harboring thrombospondin-secreting glioma with the thrombospondin receptor antagonist gabapentin decreases their proliferation (Krishna et al., 2023). The details of the relative contribution of thrombospondin-1 from glioma cells and resident astrocytes to the enhanced functional network in glioma tissue remain to be elucidated at the cellular level.

## 7 Concluding remarks

It has been known for more than half a century that microglia cause synapse stripping. New approaches such as live imaging have provided more direct evidence that microglia engulf weak synapses during the critical period of activity-dependent synaptic plasticity. In addition to microglia, astrocytes also engulf weaker synapses. Some of the molecular signals such as C1q-C3 interaction have been revealed, and aberrant deposition of the complement C4 plays a role in pathological conditions such as schizophrenia.

In addition to microglia and astrocytes, new studies suggest that OPCs also play a role in synapse remodeling by engulfing synapses at a developmental stage when microglial engulfment is less prominent, and yet the ability of OPCs to engulf synapses appears to be dependent on functional microglia. The studies illuminate the cellular complexity at the synapse beyond the tripartite synapse (Haydon, 2001) or quad-partite synapse (Schafer et al., 2013) to the current model of “synaptic quintet.” Consisting of the pre- and postsynaptic neuronal compartments, microglia, astrocytes, and OPCs (Figure 1). However, the signaling mechanisms by which OPCs interact with neighboring cells as a part of the synapse quintet to coordinately modulate neuronal synapse formation and refinement remains largely unexplored and will likely be a subject of the next few years of research. How the components of the synaptic quintet individually and collectively contribute to the overall network function in health and disease are a subject of future investigation.

## Supplementary material

SUPPLEMENTARY TABLE S1 Genes from the gene ontology term “synapse” expressed in OPCs based on published RNA-sequence data (Zhang et al., 2014; Marques et al., 2018).
